# Validation of the schema coping inventory for dysfunctional coping strategies

**DOI:** 10.3389/fpsyg.2024.1441794

**Published:** 2024-11-27

**Authors:** Simone Gazzellini, Valerio Pellegrini, Eleonora Napoli, Vanessa Ventre, Donatella Lettori, Enrico Castelli, Barbara Basile, Mauro Giacomantonio

**Affiliations:** ^1^Neuroscience Clinic Area, Neurorehabilitation Research Area, Bambino Gesù Children’s Hospital, IRCCS, Rome, Italy; ^2^Department of Social and Developmental Psychology, ‘Sapienza’ University of Rome, Rome, Italy; ^3^School of Cognitive Psychotherapy (SPC), Rome, Italy

**Keywords:** schema coping inventory, standardization, validation, maladaptive coping strategies, schema therapy

## Abstract

**Introduction:**

According to the theoretical model of Schema Therapy, each human being has basic needs that require natural satisfaction from childhood. When these emotional needs are frustrated, early maladaptive schemas (EMS) develop, leading to coping styles that are strategies to manage the pain caused by activated EMS. The study validates and standardizes the Schema Coping Inventory in the Italian population and evaluates correlations between psychological variables and the SCI.

**Methods:**

We analysed data from a community sample of 602 Italian adults, aged between 18 and 69 years, who endorsed a structured questionnaire, involving demographic information, the Italian version of the SCI, and an array of theoretically related psychological constructs.

**Results and discussion:**

Confirmative factor analysis corroborated the tridimensional structure of the SCI (Surrender, Avoidance, Overcompensation), both in terms of the overall goodness-of-fit of the model and the single items’ factor loading in the corresponding factors. The internal consistency turned out to be satisfactory. Construct validity was assessed through convergent and divergent (positive and negative) correlations with other coping style measures and psychopathological scales. Mean values and mean standard deviations are reported for the general population, for psychopathological clinical and non-pathological samples.

## Introduction

Schema therapy (ST, Young et al., 2003) is an integrative therapeutic approach that combines elements of Gestalt and attachment theories alongside emotion-focused and cognitive-behavioral strategies. The main goal of ST is to identify clients’ unmet core needs and help them fulfill them in healthier, more functional ways. When emotional core needs are frustrated in early childhood or adolescence (resulting from situations such as abuse, neglect, or dysfunctional parenting), early maladaptive schemas (EMSs) can develop. These schemas are associated with coping strategies that were initially adaptive for managing difficult or threatening situations during childhood but may become maladaptive in adulthood, perpetuating the schemas.

Patients adopt these coping strategies as a means of survival in threatening situations during childhood, where such responses may have been the most effective way to manage those circumstances. However, these behaviors often become maladaptive in the patient’s current life, serving to reinforce the schemas.

A recent meta-analysis of 33 studies (Pilkington et al., 2021) supports the theory that childhood adversity is associated with the development of EMSs in adulthood. It further indicates that individuals who recall a lack of maternal warmth or nurturing often anticipate that their emotional needs will remain unmet. Additionally, the studies by Tsouvelas et al. (2023a, 2023b), which examined children in residential care in Greece, demonstrated that schemas related to disconnection/rejection and impaired autonomy/performance domains are predictive indicators of psychopathology. The authors concluded that assessing EMSs in children is crucial to prevent the establishment of psychopathological conditions.

Three coping styles have been identified: surrender (i.e., acting as if the schema were true), avoidance (acting as if the schema has to be avoided or escaped), and overcompensation (acting as if the opposite of the schema were true). During the child’s development, such coping responses to activated schemas result in so-called ‘schema modes,’ which define the person’s momentary emotional-cognitive-behavioral state. To make a clearer distinction, EMSs can be defined as traits. In contrast, schema modes (including coping modes) describe the momentary emotional-cognitive-behavioral state of the person, and they are considered state-based constructs (Arntz et al., 2021). Coping strategies are considered over or covert responses to EMSs, activated in the “here and now” in reaction to the activation of underlying schemata. Furthermore, in contrast to EMSs, modes include more behavioral aspects. Overall, modes refer to (1) individuals’ emotional parts, the so-called child modes (i.e., feeling sad, lonely, abused, enraged, or impulsive); (2) parental introjected messages (i.e., punitive, critical, or perfectionistic inners); and (3) specific behavioral-coping responses (i.e., avoidance and overcompensation). The overcompensation coping (recently labeled as “inversion”) leads to a state in which the opposite of the EMS is felt and believed, and it is characterized by thoughts, emotions, and behaviors that serve to prove the contrary of the underlying specific schemata (Arntz et al., 2021). A previous study (Van Wijk-Herbrink et al., 2018) found a significant correlation between overcompensation and externalizing behavior.

The scientific literature on coping modes is scarce. Gulder Altuner and Yararbas (2024) found that childhood violence and illicit substance use were associated with EMSs, particularly with the coping strategies of overcompensation and avoidance. Tenore et al. (2018) focused on non-clinical subjects. They found that specific schemas (mistrust/abuse, vulnerability to harm, and high standards), modes (demanding parent), and coping styles (intra-psychic avoidance) were associated with precise peculiarities for obsessive-compulsive characteristics (washing, checking, and obsessions). In a study on temperament, Mairet et al. (2014) underline that more introverted individuals use more avoidant strategies and that the impact of EMSs on coping strategies is stronger than the influence of coping strategies on such schemas.

However, a recent international working group aims to construct a cross-cultural taxonomy of modes, also extending the theory underlying ST with new insights into emotional needs (Arntz et al., 2021). Within this framework, there has also been a reconsideration of the purpose behind the different ways of coping with EMS activation, coming up with new labels for two of those: resignation instead of surrender and inversion instead of overcompensation (Zhu et al., 2023; Wang et al., 2023). At this moment, these new insights are being empirically tested and validated in over 30 countries. Within therapy, early identification of coping strategies might be particularly helpful in addressing the unhealthy ways by which the client deals with specific EMSs.

The schema coping inventory (SCI; Rijkeboer et al., 2010; Italian translation: Basile, unpublished) is a 12-item self-report questionnaire designed to investigate dysfunctional coping styles using the Schema Theory’s theoretical model. The SCI has a three-factor structure, and it enables measuring the three main coping responses identified by the ST model, namely surrender, avoidance, and overcompensation. Unfortunately, a peer-reviewed formal validation of the constructs and structure of the SCI is still missing, and that represents a limit of past literature that the present study addresses and aims to overcome.

On samples of the psychopathological clinic and non-clinic adolescents, Van Wijk-Herbrink et al. (2018) tested the three-factor structure of the SCI, obtaining some empirical evidence for the model’s fit. Means and standard deviations on the global and single SCI factors have been provided. Internal consistency, measured by the *Cronbach’s alpha* coefficient, was higher for the clinical sample (ranging from 0.71 to 0.78) than the non-clinical sample (from 0.61 to 0.67). Significant correlations have been found between surrender and internalizing behavior problems, avoidance and internalization, overcompensation, and externalizing behavior. However, this study showed some limitations since it was carried out only on Dutch-speaking adolescent groups, a limited variety of variables were used to prove concurrent validity, and limited statistical analyses were reported to demonstrate several types of validity.

In the interest of the clinical and scientific community, we need a systematic validation study of the SCI instrument with means and standard deviation values calculated on a more general adult population, possibly linking coping strategies to psychological and psychopathological characteristics (even if a detailed theoretical dissertation of psychopathological traits of coping strategies is beyond the aims of the present study). This study was driven by the need to obtain a tool to assess coping styles in a Schema Theory framework, which could be useful both in private and public health clinics and research activities. We chose the Italian population because of the SCI-translated version that we may use with our patients, but the results of this study can be generalized to other populations (of the Western countries, at least).

To assess the presence of psychopathological distress, each participant in the present study completed the symptom checklist 90 (SCL90; Derogatis, 1994; It. transl.: Sarno et al., 2011). The aim was to provide useful statistical indexes (i.e., mean and standard deviations) for both non-pathological and pathological populations. Moreover, we hypothesize that the three coping strategies would be differently associated with psychopathological symptoms, with surrender being the most severe condition, particularly for its association with depressive status. The external validity of the SCI was determined by investigating the associations with the brief version of the COPE (Carver, 1997; It. transl.: Conti, 2010). Since avoidance is mostly employed in anxiety disorders, and particularly in social phobia (Bögels and Mansell, 2004), the Liebowitz Social Phobia Scale (LSPS; Liebowitz, 1987; It. transl.: Conti, 2010) was used to analyze the concurrent validity of the avoidance scale. We also evaluated individual optimism versus pessimism, a depressive trait linked to surrender, using the revised version of the Life Orientation Test (LOT-R; Scheier et al., 1994). The validity of the Overcompensation scale was investigated by relating it to the State–Trait Anger Expression Inventory (STAXI; Forgays et al., 1997) and the Single Item Narcissism Scale (SINS; Konrath et al., 2014), respectively, since anger and narcissism are considered an expression of overcompensation and “inversion” of EMSs (Behary, 2013). Finally, a low internal locus of control is associated with depressive symptoms and surrender style. Therefore, we administered the locus of control (LOC; Lumpkin, 1985) scale.

Therefore, the present study aimed to standardize mean and standard deviation values for population and demographic groups and validate the SCI in an Italian adult population. For this purpose, we selected independent scales measuring the theoretical construct of the SCI. Moreover, according to the previous literature (e.g., Moritz et al., 2016; Van Wijk-Herbrink et al., 2018), we hypothesize significant correlations between dysfunctional coping strategies and psychopathological symptoms, such as the association between avoidance and anxiety/phobia and depression; overcompensation and anger and narcissism; surrender and locus of control and depression.

## Method

### SCI—Italian version

The 12 items comprising the SCI (Rijkeboer et al., 2010) were translated and adapted for the Italian language according to APA standards (see Supplementary material). After the initial translation, which was made by two independent English translators, followed by a discussion and agreement on a common version, backward translation and agreement by the original author of the SCI were reached (Beaton et al., 2000). Responses to the items of this final version were given on a 7-point Likert scale ranging from 1 = totally disagree to 7 = totally agree.

### Participants

We analyzed data from a community sample of 602 Italian adults (305 females) aged between 18 and 69 years (*M*age = 33.7, *SD*age = 11.5). Participants were geographically distributed across the country (North = 48.5%, Centre = 26.2%, South = 16.9%, Islands = 8.3%). Regarding job positions, 33.6% declared themselves to be students, 47.6% to be engaged in full-time work, 14.3% to be unemployed, and 3.5% were retired or houseworkers. As for educational level, 3.3% had a lower secondary school diploma, 45.5% a high school diploma, 44% a degree, while 7.1% had a postgraduate qualification. Participants were enrolled through Prolific, a web platform for data recruiting. The sample size was established using an *a priori* power analysis designed for structural equation models (Moshagen and Erdfelder, 2016). Following the indication of Moshagen and Erdfelder (2016), we set a threshold of 0.05 for root mean square error of approximation (RMSEA), an *alpha* of 0.05, a power of 0.90, and 51 model degrees of freedom. Analysis indicated a minimum sample size of 295 participants to achieve the desired power.

The study conforms to the ethical principles of good clinical practice, the Helsinki Declaration, and complies with the current regulations. The Independent Ethics Committee of Bambino Gesù Children’s Hospital (Protocol n. 1787/2019) approved it. The informed consent was obtained when the participants were enrolled in the study.

### Measures

The following psychopathological scales have been administered in the study.

*Schema Coping Inventory (Italian version)*: In the present research, the overall scale had a mean score of 3.47 (*SD* = 0.87), while surrender, avoidance, and overcompensation were associated with mean scores of 3.04 (*SD* = 1.24), 3.21 (*SD* = 1.24), and 4.16 (*SD* = 1.04), respectively. Descriptive statistics for the three dimensions and the overall scale across variables such as sex, age, education, parental status, clinical condition, and psychotherapy are shown in Table 1. The clinical cut-off of the SCL90 General Symptomatic Index (GSI) of 60 was used to categorize participants into “non-pathological” and “psychopathological” groups.

**Table 1:** Descriptive statistics.

Variables	Surrender	Avoidance	Overcompensation	SCI_TOT	
	Mean (SD)	Mean (SD)	Mean (SD)	Mean (SD)	*N*
Sex					
Male	2.89 (1.15)	3.20 (1.17)	4.18 (1.01)	3.42 (0.82)	297
Female	3.19 (1.30)	3.22 (1.31)	4.14 (1.06)	3.52 (0.87)	305
Age					
18–25	3.32 (1.21)	3.25 (1.21)	4.42 (1.02)	3.66 (0.80)	214
26–35	3.34 (1.14)	3.43 (1.22)	4.21 (0.94)	3.65 (0.83)	130
36–45	2.84 (1.18)	3.20 (1.32)	4.04 (1.07)	3.36 (0.88)	152
45–69	2.41 (1.24)	2.89 (1.17)	3.71 (0.99)	3.00 (0.85)	106
Education					
Secondary school	2.60 (1.10)	3.39 (0.93)	4.10 (1.14)	3.36 (0.81)	20
High school	3.17 (1.24)	3.29 (1.21)	4.18 (1.06)	3.54 (0.86)	274
Degree	3.03 (1.24)	3.16 (1.30)	4.16 (1.03)	3.45 (0.90)	265
Postgraduate	2.56 (1.03)	2.94 (1.21)	4.01 (0.92)	3.17 (0.72)	43
Parents					
No	3.18 (1.22)	3.29 (1.24)	4.24 (1.02)	3.57 (0.84)	480
Yes	2.50 (1.14)	2.90 (1.21)	3.82 (1.04)	3.07 (0.87)	122
Clinical condition					
Non-pathological^a^	2.80 (1.12)	3.03 (1.16)	4.08 (1.03)	3.30 (0.80)	467
Psychopathological	3.90 (1.24)	3.84 (1.33)	4.43 (1.02)	4.06 (0.84)	135
Psychotherapy					
No	2.91 (1.21)	3.14 (1.22)	4.12 (1.03)	3.39 (0.86)	429
Yes	3.37 (1.25)	3.39 (1.29)	4.24 (1.05)	3.67 (0.88)	173
Total	3.04 (1.24)	3.21 (1.24)	4.16 (1.04)	3.47 (0.87)	602

a Based on SCL90—GSI index.

*Brief COPE* (Carver, 1997; It. transl.: Conti, 2010) is a 28-item scale, in which redundant items were dropped from the COPE (Carver et al., 1989) and only two items for each of 14 subscales were selected: Active Coping (*M* = 2.83, *SD* = 0.74, *α* = 0.75); Planning (*M* = 2.98, *SD* = 0.80, *α* = 0.81); Positive Reframing (*M* = 2.32, *SD* = 0.86, *α* = 0.82); Acceptance (*M* = 2.75, *SD* = 0.70, *α* = 0.56); Humor (*M* = 1.96, *SD* = 0.75, *α* = 0.66); Religion (*M* = 1.43, *SD* = 0.77, *α* = 0.87); Using Emotional Support (*M* = 2.27, *SD* = 0.89, *α* = 0.85); Using Instrumental Support (*M* = 2.37, *SD* = 0.87, *α* = 0.84); Self-Distraction (*M* = 2.50, *SD* = 0.79, *α* = 0.48); Denial (*M* = 1.35, *SD* = 0.62, *α* = 0.69); Venting (*M* = 2.15, *SD* = 0.76, *α* = 0.57); Substance Use (*M* = 1.24, *SD* = 0.58, *α* = 0.92); Behavioral Disengagement (*M* = 1.65, *SD* = 0.73, *α* = 0.78); Self-Blame (*M* = 2.76, *SD* = 0.78, *α* = 0.64).

*Liebowitz Social Phobia Scale* (LSPS; Liebowitz, 1987; It. transl.: Conti, 2010) is a 24-item scale employed to assess performance (13 items) and social difficulties (11 items). Fear or anxiety in the specific situation and avoidance on a 4-point scale (from ‘none/ never’ to ‘severe/usually’) were evaluated for each item separately. We computed the overall LSPS scores for each participant by summing anxiety and avoidance scores of both performance and social dimensions (*M* = 92.8, *SD* = 28.3, *α* = 0.97).

*The revised version of the Life Orientation Test* (LOT-R; Scheier et al., 1994) is a 10-item scale. Three items measure optimism, three measure pessimism, and four need fillers. Each item is scored on a 4-point scale, from ‘strongly disagree’ to ‘strongly agree’ (*M* = 30.4, *SD* = 7.25, *α* = 0.85).

*State–Trait Anger Expression Inventory* (STAXI; Forgays et al., 1997) is a multidimensional measure of anger. We administered the 10-item scale of the state anger dimensions, to which participants provided their rates on a 5-point Likert scale (*M* = 1.80, *SD* = 0.63, *α* = 0.91).

*The Single Item Narcissism Scale* (SINS; Konrath et al., 2014) is a unique item measure assessing narcissistic personality (*M* = 2.25, *SD* = 1.48). It represents a useful and reliable tool for researchers in preventing the use of longer measures. Participants were asked to rate the item on a 7-point Likert scale from 1 (not very true of me) to 7 (very true of me).

*Locus of Control* (LOC; Lumpkin, 1985) is a 6-item scale aimed to assess the individual tendency to interpret the events of one’s life as products of one’s behavior or actions (I. LOC; *M* = 3.36, *SD* = 0.71, *α* = 0.53), or to interpret them as external causes independent of one’s will (E. LOC; *M* = 3.18, *SD* = 0.71, *α* = 0.54). Participants provided their answers on a 5-point Likert scale ranging from ‘strongly disagree’ (1) to ‘strongly agree’ (5).

*Symptom Checklist 90-Revised* (SCL-90-R; Derogatis, 1994; It. transl.: Sarno et al., 2011) is a 90-item self-report inventory. Each item is scored on a 5-point Likert scale ranging from ‘completely disagree’ to ‘completely agree.’ SCL-90 provides information about 10 dimensions of psychopathological distress in the last 7 days: somatization (*M* = 1.68, *SD* = 0.63, *α* = 0.87); obsessive-compulsive (*M* = 2.09, *SD* = 0.83, *α* = 0.89); interpersonal sensitivity (*M* = 1.87, *SD* = 0.76, *α* = 0.87); depression (*M* = 2.18, *SD* = 0.89, *α* = 0.93); anxiety (*M* = 1.74, *SD* = 0.73, *α* = 0.89); hostility (*M* = 1.63, *SD* = 0.66, *α* = 0.82); phobic anxiety (*M* = 1.37, *SD* = 0.59, *α* = 0.83); paranoid ideation (*M* = 1.79, *SD* = 0.77, *α* = 0.81); psychoticism (*M* = 1.51, *SD* = 0.55, *α* = 0.81); and sleep disturbance (*M* = 2.33, *SD* = 0.93, *α* = 0.58). It is also possible to obtain a Global Score Index (GSI), which provides information about the individuals’ overall distress level (*M* = 1.81, *SD* = 0.61, *α* = 0.98).

### Data analysis

The present study aimed to investigate the factorial structure and psychometric properties of the Italian version of the SCI. To test the goodness-of-fit of the original three-factors (i.e., surrender, avoidance, and overcompensation) structure of Rijkeboer et al. (2010), we conducted a *confirmatory factor analysis* using the diagonal weighted least squares estimator (DWLS). The model fit was evaluated following the benchmarks provided by Hu and Bentler (1999). Given the sensitivity of the *chi-square* (*χ^2^*) statistic to sample size (Chen, 2007), we mainly based on values above 0.90 for the Comparative Fit Index (CFI) and Tucker-Lewis index (TLI) and on values below 0.08 for the root mean square error of approximation (RMSEA) and standardized root mean square residual (SRMR). The *CFA* was conducted using *lavaan* (Rosseel, 2012), an *R* package for structural equation modeling, using the *RStudio* graphical interface (Posit team, 2022).

To test the convergent and divergent validity, we computed correlations between the dimensions of the SCI and the Brief COPE’s distinct dimensions of active coping, planning, positive reframing, acceptance, humor, religion, using emotional support, using instrumental support, self-distraction, denial, venting, substance use, behavioral disengagement, and self-blame. The significance level of each correlation was adjusted with the Bonferroni method, given the large number of multiple tests performed. Analyses were conducted using the Psych R package (Revelle and Revelle, 2015).

Concurrent validity was first investigated by computing correlations of SCI’s dimensions with conceptually concurrent measures, such as the Liebowitz Social Phobia Scale (Liebowitz, 1987; It. transl.: Conti, 2010), Life Orientation Test (Scheier et al., 1994), state anger dimension of the State–Trait Anger Expression Inventory (Forgays et al., 1997), Single Item Narcissism Scale (Konrath et al., 2014), Locus of Control (Lumpkin, 1985) and Symptom Checklist 90-Revised (Derogatis, 1994; It. transl.: Sarno et al., 2011). Criterion validity was further investigated using a series of multiple regression models. The models included surrender, avoidance, and overcompensation as predictors and the distinct symptoms of SCL-90-R as criteria. Regression analyses aimed to further discriminate each SCI dimension and weigh their predictive power of symptomatic outcomes. Finally, we conducted *univariate ANOVAs* considering each dimension (recoded into tertiles) of the SCI as fixed factors and the total score (GSI) of the SCL-90-R as a criterion. These analyses merely provided a descriptive overview of the overall symptomatology across distinct levels of surrender, avoidance, and overcompensation.

## Results

### Confirmatory factor analysis

**Figure 1:**
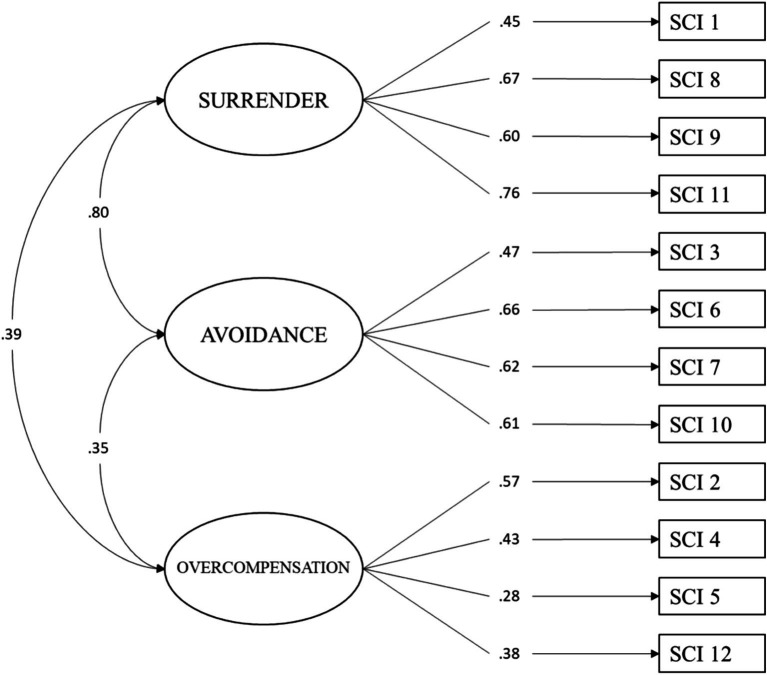
Factor structure of the Italian version of the schema coping inventory.

First, we tested the original three factors and the 12-item model of the SCI (Figure 1). The analysis revealed an acceptable model fit of this factorial structure within our community sample. Specifically, besides a significant *Chi-square* (*χ^2^* = 210.83, *df* = 51, *p* < 0.001), we found the considered incremental fit index to be over the acceptability threshold of 0.90 (CFI = 0.92). Regarding the absolute fit indices, the analyses showed a value of 0.07 for the SRMR and 0.07 for the RMSEA with a 90% confidence interval ranging from 0.06 to 0.08. These findings suggested that the model’s fit to the observed data was acceptable (Table 2). Moreover, as seen in Table 3, the factor loadings of the items were moderate to high and significant, highlighting coefficients between 0.28 and 0.76 in their standardized version.

**Table 2:** Goodness-of-fit indicators for the 3-factor and 12-item model of the Italian version of schema coping inventory.

Models	*χ^2^*	*df*	CFI	SRMR	RMSEA
Sample (*N* = 602)	210.83	51	0.92	0.07	0.07 (0.062, 0.082)

**Table 3:** Items’ factor loading for the 3-factor and 12-item model of the Italian version of the schema coping inventory.

						95%CI	
Factor	Item	*β*	*se*	*z*	*p*	Lower	Upper
Surrender	SCI_1	0.45	0.030	15.2	< 0.001	0.395	0.512
	SCI_8	0.67	0.038	17.6	< 0.001	0.592	0.741
	SCI_9	0.60	0.031	19.3	< 0.001	0.540	0.663
	SCI_11	0.76	0.037	20.6	< 0.001	0.683	0.827
Avoidance	SCI_3	0.47	0.031	15.1	< 0.001	0.409	0.531
	SCI_6	0.66	0.035	19.2	< 0.001	0.594	0.729
	SCI_7	0.62	0.035	17.7	< 0.001	0.550	0.687
	SCI_10	0.61	0.033	18.1	< 0.001	0.540	0.670
Overcompensation	SCI_2	0.57	0.056	10.1	< 0.001	0.456	0.676
	SCI_4	0.43	0.048	8.89	< 0.001	0.333	0.521
	SCI_5	0.28	0.042	6.66	< 0.001	0.197	0.361
	SCI_12	0.38	0.045	8.52	< 0.001	0.294	0.470

### Internal consistency

To assess the internal consistency of the proposed scale, we tested its reliability within the interested community sample. We also separately assessed the reliability of the three dimensions: surrender, avoidance, and overcompensation. The reliability analysis showed a satisfactory *Cronbach’s alpha* of 0.74 for the overall scale. The dimensions of surrender and avoidance yielded suitable results, with *Cronbach’s alpha* of 0.71 and 0.68, respectively. However, the dimension of overcompensation showed a low reliability coefficient (*α* = 0.47).

### Convergent and divergent validity

We computed correlations with other theoretically convergent and divergent measures to test the convergent and divergent validity of the Italian version of the SCI. More specifically, we investigated the associations of SCI’s dimensions with the distinct coping styles proposed by the Brief COPE. As shown in Table 4, we found corroboration in favor of the construct validity of SCI. The dimension of surrender highlighted convergent associations with the maladaptive coping styles of denial (*r* = 0.25, *p* < 0.001), behavioral disengagement (*r* = 0.52, *p* < 0.001), substance use (*r* = 0.19, *p* < 0.001), and self-blame (*r* = 0.31, *p* < 0.001). The avoidance dimension showed significant positive associations with denial (*r* = 0.18, *p* < 0.01), behavioral disengagement (*r* = 0.36, *p* < 0.001), and self-blame (*r* = 0.16, *p* < 0.05). Overcompensation was not significantly related to any maladaptive coping styles of the brief COPE.

**Table 4:** Intercorrelation of the schema coping inventory’s dimensions of surrender, avoidance, and overcompensation with brief cope’s dimensions, the Liebowitz Social Phobia Scale (LSPS), the Single Item Narcissism Scale (SINS), life orientation test-revised (LOT-R), trait anger (STAXI), internal (I. LOC) and external (E. LOC) locus of control.

	Surrender	Avoidance	Overcompensation
P. Reframing	−0.29***	−0.23***	0.06
S. Distraction	0.11	0.12	0.07
Venting	0.12	−0.12	0.12
U. I. support	0.05	−0.25***	−0.02
Active coping	−0.40***	−0.28***	−0.13
Denial	0.25***	0.18**	0.03
Religion	−0.11	−0.11	−0.06
Humor	−0.03	−0.001	0.03
B. Diseng.	0.52***	0.36***	0.15
U. E. Support	0.09	−0.23***	0.001
Substance Use	0.19***	0.06	0.05
Acceptance	−0.27***	−0.18**	−0.12
Planning	−0.41***	−0.26***	−0.11
Self-blame	0.31***	0.16*	0.12
SCL-GSI	0.55***	0.36***	0.21***
LSPS	0.60***	0.48***	0.13
SINS	0.10	0.12	0.30***
LOT-R	−0.64**	−0.42***	−0.19***
STAXI	0.27***	0.19***	0.22***
I. LOC	−0.41***	−0.26***	−0.09
E. LOC	0.37***	0.27***	0.18**

*p* is computed according to Bonferroni multiple tests adjustment; *p < 0.05, **p < 0.01, ***p < 0.001.

Surrender also showed significant divergent associations with ‘functional’ coping dimensions of positive reframing (*r* = −0.29, *p* < 0.001), active coping (*r* = −0.40, *p* < 0.001), acceptance (*r* = −0.27, *p* < 0.001), and planning (*r* = −0.41, *p* < 0.001). Avoidance highlighted significant and negative relations with positive reframing (*r* = −0.23, *p* < 0.001), using instrumental support (*r* = −0.25, *p* < 0.001), active coping (*r* = −0.28, *p* < 0.001), acceptance (*r* = −0.18, *p* < 0.01), and planning (*r* = −0.26, *p* < 0.001). Avoidance was the unique SCI dimension significantly associated with using emotional support (*r* = −0.23, *p* < 0.01). Overcompensation was not significantly related to functional coping styles.

### Concurrent validity

We computed correlations among each dimension of the SCI and several variables that may be framed as their potential external criteria. Then, following the indication of Eid et al. (2011) for comparing correlations from dependent samples, we also contrasted the emerged coefficients. As shown in Table 4, the Liebowitz Social Phobia Scale (LSPS) is positively related to Surrender and avoidance but not to overcompensation. The association between Surrender and LSPS was stronger than that with Avoidance (*z* = 7.41, *p* < 0.001). The three dimensions of SCI also correlated positively with anger (STAXI; see Table 4). In this case, the only relationships that differed were those with surrender and avoidance (*z* = 2.14, *p* = 0.02). As for narcissism, the analyses showed an inverse pattern: overcompensation was the unique SCI dimension to be significantly related to it. Surrender, avoidance, and overcompensation correlated negatively with the LOT. The relationship with Surrender was stronger compared to that with Avoidance (*z* = −7.11, *p* < 0.001) and Overcompensation (*z* = −10.35, *p* < 0.001). In turn, the association between LOT and Avoidance was significantly stronger than that with overcompensation (*z* = −4.77, *p* < 0.001). A similar pattern was repeated with the Internal Locus of control. Both surrender and avoidance showed a significant correlation with the Internal Locus of control, with Surrender showing a significantly larger coefficient than avoidance (*z* = −4.18, *p* < 0.001), while overcompensation was not significantly related to the internal locus of control. As for the external locus of control, all the dimensions of SCI showed a positive relationship. However, surrender showed a higher coefficient than the other two (*z* = 1.92, *p* = 0.03; *z* = 3.24, *p* < 0.001, respectively, for avoidance and overcompensation), which were also different from each other (*z* = 1.80, *p* = 0.04). Finally, each dimension of SCI correlated positively with psychopathological symptomatology measured with SCL-90-R. Surrender had a stronger association with psychopathological symptoms than avoidance (*z* = 5.72, *p* < 0.001) and overcompensation (*z* = 7.47, *p* < 0.001). In turn, avoidance had a significantly stronger relation to symptomatology than overcompensation (*z* = 3.07, *p* < 0.001). To provide an overview of the psychopathological symptomatology for different levels of the three dysfunctional coping styles, we also implemented a series of *ANOVAs* (see Supplementary material).

### Multiple regression analyses

To further examine the criterion validity of SCI, we implemented a multiple regression model for each symptom dimension of the SCL-90-R (considered as a criterion) and the surrender, avoidance, and overcompensation dimensions as predictors. For the symptoms dimension of somatization, we found surrender as a unique significant predictor (*β* = 0.33, *se* = 0.046, *z* = 7.06, *p <* 0.001, 95%CI = 0.234, 0.415). Both surrender (*β* = 0.52, *se* = 0.041, *z* = 12.8, *p <* 0.001, 95%CI = 0.440, 0.600) and overcompensation (*β* = 0.11, *se* = 0.035, *z* = 3.17, *p =* 0.002, 95%CI = 0.042, 0.117) turned out to be positively associated with obsessive-compulsive symptoms. As for interpersonal sensitivity, all three of the SCI’s dimensions of Surrender (*β* = 0.44, *se* = 0.041, *z* = 10.9, *p <* 0.001, 95%CI = 0.362, 0.522), avoidance (*β* = 0.14, *se* = 0.041, *z* = 3.53, *p <* 0.001, 95%CI = 0.064, 0.223), and overcompensation (*β* = 0.06, *se* = 0.035, *z* = 3.17, *p =* 0.002, 95%CI = 0.042, 0.117) simultaneously resulted as significant predictors. Depression symptoms was positively related only with surrender (*β* = 0.53, *se* = 0.040, *z* = 13.0, *p <* 0.001, 95%CI = 0.446, 0.605). The anxiety dimension of SCL90-R was predicted by both surrender (*β* = 0.47, *se* = 0.043, *z* = 11.0, *p <* 0.001*,* 95%CI = 0.385, 0.553) and overcompensation (*β* = 0.08, *se* = 0.036, *z* = 2.17, *p* = *0*.031, 95%CI = 0.007, 0.150), as well as Hostility (*β* = 0.25, *se* = 0.046, *z* = 5.40, *p <* 0.001, 95%CI = 0.158, 0.338; *β* = 0.22, *se* = 0.039, *z* = 5.72, *p <* 0.001, 95%CI = 0.147, 0.300; respectively, for surrender and overcompensation). Phobic anxiety symptomatology was instead predicted by the dimensions of surrender (*β* = 0.35, *se* = 0.045, *z* = 7.82, *p <* 0.001, 95%*CI* = 0.147, 0.325) and avoidance (*β* = 0.11, *se* = 0.045, *z* = 2.42, *p* = *0*.016, 95%CI = 0.020, 0.196). Paranoid ideation and psychoticism were both positively associated with all three dimensions of SCI (see Table 5). Finally, surrender only predicted sleep disturbance (*β* = 0.29, *se* = 0.047, *z* = 6.15, *p <* 0.001, 95%*CI* = 0.198, 0.385).

**Table 5:** Multiple regression of surrender, avoidance, and overcompensation dimensions on SCL-90 symptomologies.

						95%CI	
Criterion	Predictor	*β*	*se*	*z*	*p*	Lower	Upper
SOM	Surrender	0.33	0.046	7.06	0.001	0.234	0.415
	Avoidance	0.04	0.046	0.76	0.45	−0.056	0.125
	Overcompensation	0.05	0.039	1.37	0.17	−0.023	0.130
O-C	Surrender	0.52	0.041	12.8	0.001	0.440	0.600
	Avoidance	0.03	0.041	0.63	0.53	−0.054	0.105
	Overcompensation	0.11	0.035	3.17	0.002	0.042	0.177
INT	Surrender	0.44	0.041	10.9	0.001	0.362	0.522
	Avoidance	0.14	0.041	3.53	0.001	0.064	0.223
	Overcompensation	0.10	0.035	2.97	0.003	0.035	0.171
DEP	Surrender	0.53	0.040	13.0	0.001	0.446	0.605
	Avoidance	0.06	0.040	1.43	0.15	−0.022	0.137
	Overcompensation	0.06	0.034	1.07	0.08	−0.009	0.126
ANX	Surrender	0.47	0.043	11.0	0.001	0.385	0.553
	Avoidance	0.01	0.043	0.29	0.77	−0.071	0.096
	Overcompensation	0.08	0.036	2.17	0.031	0.007	0.150
HOS	Surrender	0.25	0.046	5.40	0.001	0.158	0.338
	Avoidance	−0.003	0.046	−0.07	0.94	−0.093	0.087
	Overcompensation	0.22	0.039	5.72	0.001	0.147	0.300
PHOB	Surrender	0.35	0.045	7.82	0.001	0.262	0.438
	Avoidance	0.11	0.045	2.42	0.016	0.020	0.196
	Overcompensation	−0.009	0.038	−0.23	0.82	−0.083	0.066
PAR	Surrender	0.24	0.045	5.20	0.001	0.147	0.325
	Avoidance	014	0.045	3.15	0.002	0.054	0.232
	Overcompensation	0.14	0.039	3.67	0.001	0.066	0.217
PSY	Surrender	0.35	0.043	8.19	0.001	0.269	0.439
	Avoidance	0.15	0.043	3.47	0.001	0.065	0.235
	Overcompensation	0.08	0.037	2.14	0.033	0.006	0.151
SLEEP	Surrender	0.29	0.047	6.15	0.001	0.198	0.385
	Avoidance	−0.06	0.047	−1.17	0.24	−0.148	0.038
	Overcompensation	0.01	0.040	0.35	0.72	−0.065	0.093

Summary of explained variance for each multiple regression model: Somatization (SOM): *R*^2^ = 0.13; Obsessive-Compulsive (O-C): *R*^2^ = 0.32; Interpersonal Sensitivity (INT): *R*^2^ = 0.32; Depression (DEP): *R*^2^ = 0.33; Anxiety (ANX): *R*^2^ = 0.25; Hostility (HOS): *R*^2^ = 0.13; Phobic Anxiety (PHOB): *R*^2^ = 0.18; Paranoid Ideation(PAR): *R*^2^ = 0.15; Psychoticism(PSY): *R*^2^ = 0.23; Sleep Disturbance (SLEEP): *R*^2^ = 0.07.

## Discussion

In the present research, using a large community sample (*N* = 602), we provide empirical corroboration in favor of the factorial structure and psychometric properties of the Schema Coping Inventory (SCI-Italian version; Rijkeboer et al., 2010). SCI measures dysfunctional strategies to cope with maladaptive early schemas. Such coping mechanisms refer to how a person deals with an internal experience (schema activation) rather than an external circumstance (Arntz et al., 2021). Means and standard deviations (for SCI and all the psychopathological questionnaires), confirmatory factor analysis, internal consistency, construct validity, and criterion validity analyses have been reported.

The factor analysis corroborated the tridimensional structure of the SCI, i.e., Surrender, Avoidance, and Overcompensation, both in terms of the overall goodness-of-fit of the model and the single items’ factor loading in the corresponding factors. The *internal consistency* of the whole scale turned out to be satisfactory. Regarding the reliability of each of the three dimensions, we found adequate coefficients associated with the dimensions of Surrender and Avoidance, while the dimension of Overcompensation showed a low reliability coefficient. Unfortunately, comparing this data with previous studies is impossible (Rijkeboer et al., 2010; Van Wijk-Herbrink et al., 2018). Convergent and divergent validity have been assessed by correlating the SCI scales with other measures indexing coping styles and psychopathological features.

The dysfunctional Surrender coping style of the SCI (SCI-surrender) significantly and positively correlated with other Brief COPE dimensions associated with depressive and surrender styles, i.e., denial, behavioral disengagement, self-blame, self-distraction, and substance use. In addition, SCI-surrender was negatively related to the ‘functional’ coping dimensions of positive reframing, active coping, acceptance, and planning (Carver, 1997). These data may be interpreted as evidence of multiple components in surrender, representing disengagement strategies (denial, self-distraction, and substance use) and/or responses to self-blame (Janovsky et al., 2023). All of them would work by blocking functional, active coping, such as planning, positive reframing, and, finally, hindering acceptance mechanisms. Notably, self-blame, together with behavioral disengagement and denial, represents significant components associated with avoidance behavior as well. SCI-avoidance showed moderately significant and negative correlations with use of instrumental support and use of emotive support, suggesting a specific reduction of social support searching in the case of avoidance (Polman et al., 2010; Forbes et al., 2020), whereas their correlations with surrender and overcompensation were null or weak. This evidence suggests that the use of an avoidance coping strategy is associated with a reduced use and expectation of different types of support: emotive and instrumental. Several significant correlations emerged between the three SCI factors and other psychopathological dimensions (Janovsky et al., 2023). By contrasting correlation coefficients of interest, we found that surrender showed a significantly higher correlation with LSPS (Derogatis, 1994), measuring fear and anxiety in performance and social exposure than avoidance and overcompensation, which are significantly associated with social phobia (Beidel and Randall, 1994; Gonzalez Diez et al., 2012). Surrender-LSPS association would sound contrary to expectations and suggests a possible pathological generalization of the surrender strategy even to anxiety disorders. Surrender and avoidance were associated with anger, whereas overcompensation showed the strongest correlation with narcissism, as hypothesized (Rosenthal and Hooley, 2010; Behary, 2013; Young et al., 2003). As was found in earlier research (Van Wijk-Herbrink et al., 2018), overcompensation was mainly related to externalizing tendencies, as it was associated with, for example, narcissism and hostility. However, since we did not find significant correlations between overcompensation and other brief COPE factors, we suggest that a more defined operationalization and theoretical agreement on the construct should be achieved.

As predicted, the values on the LOT-R scale, assessing aptitude for optimism and pessimism, especially individual pessimism (Scheier et al., 1994), were more strongly correlated with the surrender style than the other two styles. We found the same pattern for the correlations with locus of control (see also Reinert, 1997). These results might be related to the belief people can have in perceiving themselves as less capable and skilled in facing the schema, feeling as if they are “a child” in a world defined by the representation of their underlying schema (Arntz et al., 2021). Regarding the overall correlations with SCL-90 GSI, surrender showed the strongest correlation with the psychopathological global index, followed by avoidance and overcompensation, respectively. The evidence confirms the initial hypothesis of differential psychopathological sequela for the three coping strategies. Surrender was the dysfunctional coping style that was more strongly associated with the presence of psychopathological mechanisms, i.e., SCL-90 psychopathological global index and specific measures, such as social phobia, anger, pessimism, and polarization of internal and external locus of control attribution. Surrendering as a coping strategy tends to develop earlier in childhood compared to overcompensation, particularly in highly dysfunctional contexts. As a result, it may lead to more severe and pervasive early maladaptive schemas (EMSs).

As the *multiple regression analysis* showed, SCI criterion validity has been investigated using the psychopathological dimensions of the SCL-90-R. The surrender strategy proved to be exclusive in explaining the variability of the dimensions of depression, somatization, and sleep disturbance. Therefore, surrender is attested to be the coping strategy associated with mood depression and its related symptoms. Surrender and overcompensation were significantly related to the ‘anxiety’ dimensions of obsessive-compulsive, hostility, and anxiety (see also Tenore et al., 2018). Surrender and avoidance significantly explained phobic symptomatology, which is typically maintained by emotional and behavioral avoidance.

On the other hand, all three coping strategies were significantly related to the dimensions of paranoid ideation, psychoticism, and interpersonal sensitivity. As we can observe, the surrender factor is a good predictor for each psychopathological dimension of the SCL-90 scale (see also Starcevic et al., 2015). The SCI validity is supported by the significant correlations with the COPE brief factors: once again, surrender showed strong correlations with dysfunctional coping strategies. Therefore, surrender coping is giving in to EMS, which will lead to vulnerable child modes, and it is more related to psychopathological features (i.e., depression, anxiety, OCD, and so on) with respect to avoidance and overcompensation.

In the schema therapy theory, Surrender is hypothesized to lead to, for example, the vulnerable child modes in which intense distress is experienced (Arntz et al., 2021; Sójta and Strzelecki, 2023). As expected, Surrender had the strongest associations with psychopathological symptoms as measured by the SCL-90, such as depression, anxiety, obsessive-compulsive symptoms, somatization, and sleep problems. The hypothesized function of Avoidance is to escape the pain of the EMSs. In terms of overcompensation to deny the pain by assuming the opposite, in line with these functions, hardly any associations between avoidance or overcompensation and the symptoms indexed by the SCL-90 LSPS were found.

The results of our study are in line with the findings by Van Wijk-Herbrink et al. (2018), that is, there is a significant association between dysfunctional coping styles and psychopathological traits. However, we overcome the limitations of data analysis and generalization found in the study by Van Wijk-Herbrink et al. (2018), which was conducted solely on a Dutch adolescent group consisting of both community and clinical samples with externalizing or personality disorders.

In our opinion, a clinically relevant limitation of the SCI questionnaire is the absence of a dimensional measure of a positive and functional coping strategy, which would allow classifying individuals’ behaviors even in an ‘adaptive functional’ profile, in line with previous tools, such as COPE Brief. Previous studies demonstrated that the use of positive coping strategies is correlated with a better quality of life and better capacity for resilience in people with psychiatric disorders (Chi et al., 2022; Tsouvelas et al., 2023a, 2023b). On the contrary, the notable strength of the SCI is the reduced number of items and factors, which is useful in both research and clinical application.

In our opinion, SCI classification in surrender, avoidance, and overcompensation catches and summarizes the variety of dysfunctional coping behaviors in response to schema activation. In particular, the construct of ‘overcompensation’ highlights the importance of the ‘quantity’ dimension of a class of behaviors that may be functional and adaptive until a certain quantitative threshold but become dysfunctional beyond such a threshold. Such a hypothesis should be verified in future research. However, overcompensation, as defined right now, may be subject to several theoretical interpretations, and a more detailed operationalization would be helpful.

### Limitations and conclusion

Some limitations need to be acknowledged. A first limitation could be found in the use of a paid platform for the recruitment of participants. While Prolific is a highly effective participant recruitment platform, it has some limitations that can impact the quality of the collected data. One major issue is uncertainty about participant identity. Despite Prolific’s verification processes, it is not always possible to ensure that participants do not misrepresent their demographic data or update it inconsistently over time. Another potential issue is the lack of control over participants’ environments. It is difficult to control for variables such as distractions, the type of device used, or the environment the participant is in while completing the questionnaire. This is a disadvantage compared to procedures carried out in controlled laboratory environments, where researchers can supervise participants in real time. However, Prolific stands out as a valuable alternative to other crowd-working platforms due to its clear guidelines for participants and researchers. One of its key advantages is transparency. Participants are fully informed that they are being recruited for research, and they understand the details regarding payment, treatment, and their rights and responsibilities within this context. For researchers, Prolific offers greater transparency about the participant pool than other platforms, allowing for more thorough screening based on various criteria before selecting participants for their studies (Palan and Schitter, 2018).

The sample may not fully represent the broader Italian population due to platform recruitment, particularly regarding socioeconomic or cultural diversity. Future research could address this limitation with a more diverse or clinically representative sample. However, data collection was implemented with accurate attention checks, which, if failed, led to the automatic exclusion of the participants and the non-registration of the related data. Thus, we obtained reliable data on which to base our analysis and the related evidence.

Another limitation might be traced to the low-reliability value of the overcompensation dimension of SCI, which mirrors the need for a more detailed theoretical definition of the construct and for the development of a new psychometrically robust instrument to measure dysfunctional coping styles derived from schema therapy-associated theory.

We can conclude that having measures describing the dysfunctional coping styles for psychological pain and stress allows us to (1) increase the patient’s awareness of his/her own coping style; (2) increase the patient’s awareness of other possible coping styles for stress and pain; (3) plan therapeutic actions addressing the dysfunctional style, aiming at modifying it, enlarging strategies’ variability, and hence working on cognitive flexibility, suggesting more functional strategies; and (4) increase awareness and acceptance of different coping styles into the framework of interpersonal systems, i.e., couple, family, clinical or rehabilitative pediatric team, professional team.

## Data Availability

The raw data supporting the conclusions of this article will be made available by the authors without undue reservation.
