# Supplementary material for: Validation of the schema coping inventory for dysfunctional coping strategies
Source: Front Psychol. 2024 Nov 27;15:1441794. doi: 10.3389/fpsyg.2024.1441794 (PMC11633441; doi:10.3389/fpsyg.2024.1441794)
Supplement: Supplementary file 1 [file Data_Sheet_1.docx]

**Additional Analysis**

**Univariate ANOVAs**

As a complement to our main research finding, we conducted three univariate ANOVAs aimed at providing an overview of the global distress across different levels of each SCI dimension. To conduct the analyses, we recoded Surrender, Avoidance, and Overcompensation into tertiles and considered them as fixed factors. The criterion variable was instead represented by the GSI of the SCL-90-R. As expected, analyses revealed a main effect of Surrender (*F*_(1, 600)_ = 95.6, *p* < .001), Avoidance (*F*_(1, 600)_ = 31.4, *p* < .001), and Overcompensation (*F*_(1, 600)_ = 9.66, *p* < .001). As shown in Table 6, *Bonferroni post hoc comparisons* highlighted significant differences in the overall distress across low, medium, and high levels of Surrender. A significant difference in levels of symptomatology was found for high vs medium and high vs low levels of Avoidance, but no difference was found between medium and low levels of Avoidance, while only differences in distress were found between high and low levels of Overcompensation (see Figure 2).

Table S1. *Post hoc comparisons between low, medium, and high tertiles of Surrender (SURR), Avoidance (AVOI), and Overcompensation (OVER) on the global score of SCL-90-R*

| *SCI* | *Comparison* | *M (SD)* | *M (SD)* | *Diff.* | *se* | *p* | *95%CI* | |
| --- | --- | --- | --- | --- | --- | --- | --- | --- |
|  |  | *(i)* | *(j)* | *(i–j)* |  |  | *Lower* | *Upper* |
| SURR | High vs Low | 2.22 (.68) | 1.47 (.44) | 0.75 | .06 | .001 | .621 | -.884 |
|  | High vs Med. | 2.22 (.68) | 1.77 (.46) | 0.45 | .05 | .001 | .320 | .574 |
|  | Med. vs Low | 1.77 (.46) | 1.47 (.44) | 0.31 | .05 | .001 | .180 | .432 |
| AVOI | High vs Low | 2.06 (.68) | 1.63 (.55) | 0.43 | .06 | .001 | .298 | .569 |
|  | High vs Med. | 2.06 (.68) | 1.74 (.50) | 0.32 | .06 | .001 | .180 | .464 |
|  | Med. vs Low | 1.74 (.50) | 1.63 (.55) | 0.11 | .06 | .18 | -.030 | .253 |
| OVER | High vs Low | 1.95 (.62) | 1. 68 (.55) | 0.27 | .06 | .001 | .122 | .416 |
|  | High vs Med. | 1.95 (.62) | 1.81 (.62) | 0.14 | .06 | .063 | -.005 | .278 |
|  | Med. vs Low | 1.81 (.62) | 1. 68 (.55) | 0.13 | .06 | .087 | -.013 | .278 |

Figure S1. *Mean scores of SCL-90-R across low, medium, and high tertiles of Surrender (SURR), Avoidance (AVOI), and Overcompensation (OVER)*

| 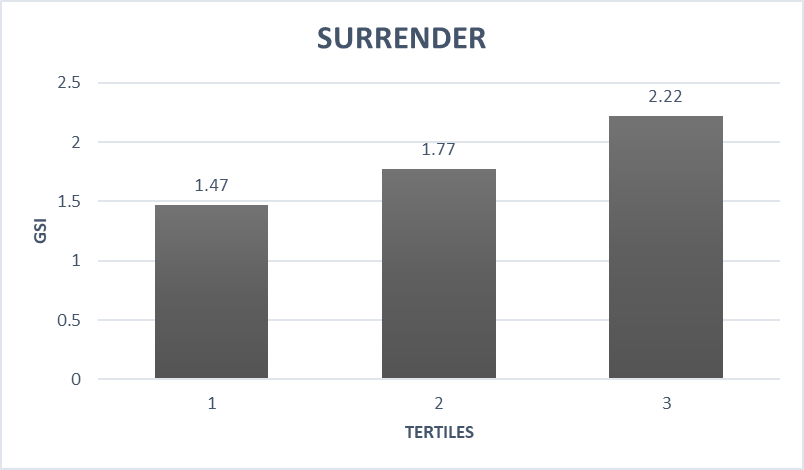 |
| --- |
| 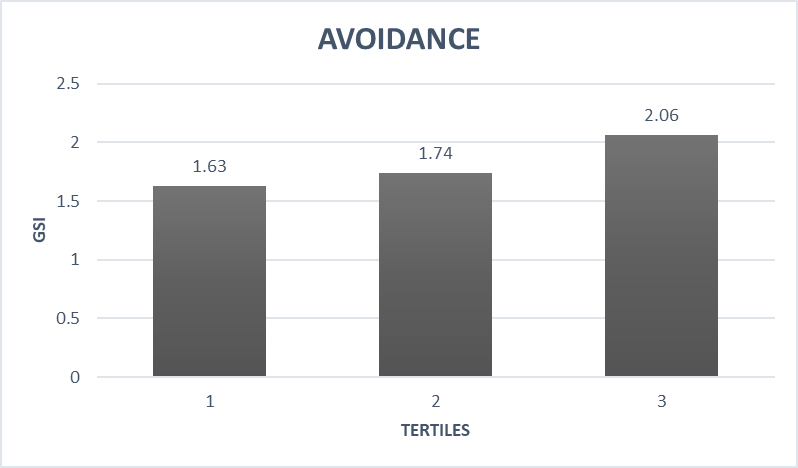 |
| 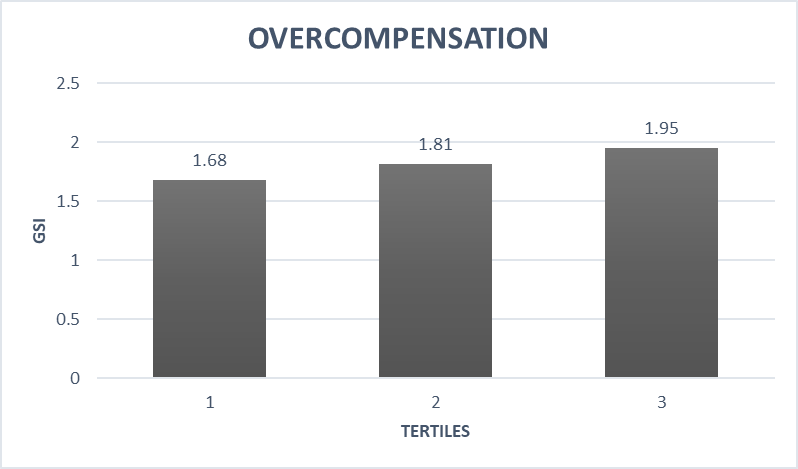 |
