# Supplementary material for: Validation of the schema coping inventory for dysfunctional coping strategies
Source: Front Psychol. 2024 Nov 27;15:1441794. doi: 10.3389/fpsyg.2024.1441794 (PMC11633441; doi:10.3389/fpsyg.2024.1441794)
Supplement: Supplementary file 2 [file Data_Sheet_2.pdf]

Appendix 1

Original English versions of the SCI.

Schema Coping Inventory

Rijkeboer, M.M., Lobbestael, J., Arntz, A., & Genderen, H. van (2010)

Instruction

Listed below are statements that people might use to describe themselves. Please read each statement, then rate it based on how accurately it fits you. When you are not sure, base your answer on what you *emotionally feel*, not on what you think to be true. Then place a circle around the figure that best describes you.

How accurately do the statements below fit you?	1= completely untrue of me<br>2= mostly untrue of me<br>3= slightly untrue of me<br>4= neither untrue, nor true<br>5= slightly true of me<br>6= mostly true of me<br>7= completely true of me
1. If others treat me bad, I let that happen.	1 2 3 4 5 6 7
2. If I get criticized, I jump to my defense.	1 2 3 4 5 6 7
3. I rather not have intimate friendships or relationships.	1 2 3 4 5 6 7
4. I can be very critical about what others do or don't do.	1 2 3 4 5 6 7
5. I tend to overrule and control others.	1 2 3 4 5 6 7
6. I prefer to avoid confrontation.	1 2 3 4 5 6 7
7. I like to keep it superficial.	1 2 3 4 5 6 7
8. I let others determine my life.	1 2 3 4 5 6 7
9. In case of trouble or difficulty I think: "You see, this has to happen to me again".	1 2 3 4 5 6 7
10. It is best to switch off your feelings as much as possible.	1 2 3 4 5 6 7
11. In case of difficulty, I tend to give up.	1 2 3 4 5 6 7
12. I fantasize to become famous, rich, important, or successful.	1 2 3 4 5 6 7

Appendix 2

Italian versions of the SCI.

Schema Coping Inventory

Gazzellini S, Pellegrini V, Napoli E, Ventre V, Lettori D, Castelli E, Basile B & Giacomantonio M (2024*, Frontiers in Psychology*)

Età______ Genere _____ Anni di istruzione _____

Istruzioni

In seguito, sono elencate alcune affermazioni che possono descrivere le persone. Leggi ciascuna di queste e indica, in base al punteggio, quanto ogni affermazione è vera per te. Quando non sei sicuro, valuta la tua risposta in base a come *ti senti emotivamente*, e non in base a cosa pensi sia vero o giusto. Per ciascuna affermazione indica con una X la risposta che meglio ti descrive.

Quanto accuratamente queste affermazioni ti descrivono?

1 = completamente falso per me
2 = abbastanza falso per me
3 = leggermente falso per me
4 = né falso né vero per me
5 = leggermente vero per me
6 = abbastanza vero per me
7 = completamente vero per me

1	Se gli altri mi trattano male, permetto che questo accada	1	2	3	4	5	6	7
2	Se vengo criticato, scatto subito sulla difensiva	1	2	3	4	5	6	7
3	Preferisco non avere relazioni amicali o sentimentali troppo intime	1	2	3	4	5	6	7
4	Posso essere molto critico rispetto a quello che gli altri fanno o non fanno	1	2	3	4	5	6	7
5	Ho la tendenza a condizionare e prevaricare sugli altri	1	2	3	4	5	6	7
6	Preferisco evitare di confrontarmi	1	2	3	4	5	6	7
7	Mi piace restare a livello più superficiale	1	2	3	4	5	6	7
8	Lascio che siano gli altri a decidere della mia vita	1	2	3	4	5	6	7
9	In caso di problemi o difficoltà penso: "Vedi, è quello che mi succede sempre!"	1	2	3	4	5	6	7
10	È meglio sopprimere il più possibile le emozioni	1	2	3	4	5	6	7
11	Nelle situazioni di difficoltà, tendo ad arrendermi	1	2	3	4	5	6	7
12	Fantastico sul diventare famoso, ricco, importante o di successo	1	2	3	4	5	6	7
